# Reasons for Retraction of Biomedical Articles Written by Eastern Mediterranean and Turkish Authors; A Comprehensive Cross-Sectional Study During 2010-2019: Short title: Reasons for Retraction of Biomedical Articles

**DOI:** 10.31661/gmj.v15i.3885

**Published:** 2026-01-30

**Authors:** Kezhal Bijari, Mehrdad Askarian, Mehrdad Askarian

**Affiliations:** ^1^ Department of Medical Journalism, School of Paramedical Sciences, Shiraz University of Medical Sciences, Shiraz, Iran; ^2^ Department of Community Medicine, School of Medicine, Shiraz University of Medical Sciences, Shiraz, Iran; ^3^ Department of Health Research Methods, Evidence and Impact, Faculty of Health Sciences, McMaster University, Hamilton, Ontario, Canada

**Keywords:** Article Retraction, Authorship, Plagiarism, Retracted Article, Retractions, Scientific Misconduct

## Abstract

**Background:**

Article retraction means removing a published article from the journal because of ethical issues or scientific errors in order to correct the literature. In this study, we aimed to determine the reasons for retracting biomedical articles written by authors from Iran, Saudi Arabia, Pakistan, Egypt, and Turkey.

**Materials and Methods:**

This cross-sectional study included all retracted biomedical articles with first authors affiliated with Iran, Saudi Arabia, Pakistan, Egypt, or Turkey, retracted between September 1, 2010, and September 1, 2019. Data were extracted from Retraction Watch, MEDLINE, PubMed Central (PMC), Clarivate Analytics, and Scopus. Each article’s information was entered into a data collection form and analyzed using SPSS version 24.

**Results:**

Of 436 retracted articles, Iran had the highest number (223), followed by Turkey (80), Egypt (72), Saudi Arabia (35), and Pakistan (26). Common causes of retraction included plagiarism, duplication, authorship issues, and fake peer review. In Iran, fake peer review (42.6%) and authorship issues (41.3%) were most prevalent. Significant inter- country differences were found in retraction frequency and causes. The most affected fields were biology, biochemistry, oncology, cardiovascular, surgery, and pathology.

**Conclusion:**

The results showed that scientific misconducts (plagiarism, duplication, authorship issues, and fake peer review) were the main reasons for retracting the articles in the five studied countries. To reduce such misconducts, regional regulatory policies, improved editorial practices, and enhanced research ethics training are urgently needed.

## Introduction

Retraction is the removal of a published article due to ethical concerns or scientific errors, aiming to correct the literature and alert readers to unreliable findings. This issue poses a significant challenge in biomedical research [1]. In 2009, the Committee on Publication Ethics (COPE) issued guidelines outlining key reasons for retraction, including misconduct, honest errors, redundant publication, plagiarism, and unethical research [1].

The increasing number of article retractions has raised concerns among researchers and editors. This rise is due to more low-quality papers being published and editors becoming more willing to correct the literature [2]. It reflects systemic issues like the "publish-or-perish" culture, which values quantity over quality [3]. Higher retraction rates also threaten scientific integrity by reducing public trust, wasting resources, and complicating clinical decision-making [4].

COPE plays a key role in guiding editors to address ethical misconducts [5]. While most studies are honest, some face retraction due to errors or misconduct. Honest mistakes are inevitable but can harm public trust. Scientific misconducts, though rarer, are more serious and include plagiarism, data falsification, and ethical violations [6][7].

In the Eastern Mediterranean region, the structural context of research presents unique challenges that make studying article retraction important. Limited funding opportunities, lack of national ethics guidelines, and disparities in research governance across countries have contributed to inconsistent research practices and oversight. According to the World Health Organization (WHO) reports, many institutions in the region lack sustainable systems for ethics review and grant management, which may increase the risk of both honest errors and misconduct [8]. Therefore, understanding retraction trends in this region is essential for improving research integrity and guiding future policy development.

Although biomedical publications have grown quickly in the Eastern Mediterranean region, progress in research oversight and infrastructure has not kept pace. Resource limitations, fragmented ethics governance, and inconsistent peer review practices raise concerns about research integrity and increase the likelihood of article retraction [8]. Despite this, to the best of our knowledge, no comprehensive study has simultaneously investigated multiple databases to examine the reasons for article retraction among Iranian authors and compared Iran to other countries in the region. Therefore, we conducted this study to determine the frequency and causes of biomedical article retractions in Iran, Saudi Arabia, Pakistan, Egypt, and Turkey.

## Materials and Methods

In this cross-sectional study, we included all retracted biomedical articles published between September 1, 2010, and September 1, 2019, whose first author was affiliated with Iran, Saudi Arabia, Pakistan, Egypt, or Turkey. These countries were selected because they share similar research conditions, including rapid growth in biomedical publications driven by institutional incentives, limited funding, academic pressures for career advancement, and weak peer review systems, such as poor reviewer training and risks from paper mills. Additionally, English is not the native language in these countries, which may influence how research is written, reviewed, and interpreted in international journals.

The selected time frame provides a consistent decade of data while avoiding potential bias introduced by the COVID-19 pandemic, which may have affected retraction patterns after 2019. We focused on first-author affiliations because the first author is typically the primary contributor to the research and responsible for manuscript preparation. This approach allows for clearer attribution of country origin and minimizes ambiguity in multi-author or cross-national collaborations.

Information on retracted articles was extracted from five databases: Retraction Watch, MEDLINE, PubMed Central (PMC), Clarivate Analytics, and Scopus. Only fully retracted articles accompanied by an official retraction notice were included; partial retractions (e.g., image removal) or cases lacking a clear retraction reason were excluded.

The search was conducted in the Retraction Watch database using country filters (Iran, Saudi Arabia, Pakistan, Egypt, and Turkey) and a defined time frame (September 1, 2010 to September 1, 2019). Retrieved articles were screened for eligibility, and relevant data were entered into a structured Excel form with five country-specific sheets. Each entry included: article title, first author’s name and affiliation, publication and retraction year, journal name, indexing databases, research field, and retraction reason.

Retraction reasons and research fields were categorized according to Retraction Watch classifications. When categories overlapped, we followed the database’s definitions. Since the Retraction Watch taxonomy does not explicitly define plagiarism and duplication, we used the COPE guidelines to distinguish them: Plagiarism refers to the unattributed use of text, ideas, or data from another source, while duplication refers to publishing substantially similar content (text, data, or figures) in more than one article by the same authors without proper cross referencing. Cases were classified based on the wording of the official retraction notices. The full taxonomy of other retraction categories is available in Appendix B of the Retraction Watch Database User Guide [9].

To verify bibliographic details and indexing coverage, each article title was cross-checked in Scopus, Clarivate Analytics, and PubMed. When discrepancies arose, such as a retraction listed in Retraction Watch but absent from PubMed, we relied on the official retraction notice from the journal or publisher. Articles lacking such confirmation were excluded to ensure consistency and data integrity.

Finally, a secondary search was performed to ensure that all the retracted articles were retrieved and nothing was missed. For this purpose, we used the advanced search section of PubMed. In the "All Fields" section, the terms "retracted publication" and "retraction of publication", were used and in the "Date-Publication" section, the time period was mentioned, and in the "Affiliation" section, the name of one of the five countries was entered, and the search was done.

Clarivate Analytics database also provides access to retracted articles through advanced search. The terms TI =retract or TI=retraction, AD =one of the five countries, and WC =the field of research were entered, and also the period of time from 2010 to 2019 was chosen.

Also, in the Scopus database, in the advanced search section, by searching "retraction note to", "retraction", or "retracted article" in "ALL" section, and by searching the names of the countries mentioned in the "AFFILCOUNTRY " section, and also by selecting the time period, the retracted articles were retrieved.

### Statistical Analysis

After completing the data collection form, the data were entered into IBM SPSS Statistics software, version 24 (IBM Corp., Armonk, NY, USA), and analyzed. The descriptive data were reported by frequency (percentage), and the percentage of the retracted articles was compared between the different countries using the Chi-square test and Fisher’s exact test. The P values less than 0.05 were considered significant.

### Ethical Considerations

The data of the retracted articles were analyzed anonymously, and the protocol of the study was approved by the Ethics Committee of Shiraz University of Medical Sciences (code: IR.SUMS.REC.1398.1013).

## Results

**Table:** Table1. The Frequency of Reasons for Articles Retraction for Each Studied Country, 2010-2019

**Reason of articles retraction**	**Iran** **N (%)**	**Egypt** **N (%)**	**Pakistan** **N (%)**	**Saudi Arabia** **N (%)**	**Turkey** **N (%)**	**P value**
**Plagiarism of text**	63 (28.3%)	22 (30.6%)	9 (34.6%)	11 (31.4%)	14 (17.5%)	0.252
**Duplication of article**	54 (24.2%)	17 (23.6%)	5 (19.2%)	11 (31.4%)	29 (36.3%)	0.204
**Concern about authorship, Lack of approval from author/s, Forged authorship **	92 (41.3%)	8 (11.1%)	2 (7.7%)	5 (14.3%)	6 (7.5%)	<0.001
**Fake peer review**	95 (42.6%)	1 (1.4%)	8 (30.8%)	0 (0%)	1 (1.3%)	<0.001
**Concerns about data, Error in data, Unreliable data**	25 (11.2%)	18 (25%)	1 (3.8%)	6 (17.1%)	11 (13.8%)	<0.001 ^†^
Misconducts by author*	49 (22%)	3 (4.2%)	0 (0%)	2 (5.7%)	5 (6.3%)	<0.001
**Concerns about results, Error in results, Unreliable results **	22 (9.9%)	11 (15.3%)	0 (0%)	3 (8.6%)	12 (15%)	0.173
**Ethical violations by author/ Breach of journal policy by author **	21 (9.4%)	4 (5.6%)	1 (3.8%)	3 (8.6%)	7 (8.8%)	0.775
**Error in methods or materials**	9 (4%)	7 (9.7%)	0 (0%)	0 (0%)	7 (8.8%)	0.065
**Error in analyses**	6 (2.7%)	3 (4.2%)	0 (0%)	0 (0%)	6 (7.5%)	0.157
**Plagiarism of image or data**	6 (2.7%)	1 (1.4%)	2 (7.7%)	1 (2.9%)	3 (3.8%)	0.582
**Duplication of image or data**	5 (2.2%)	6 (8.3%)	0 (0%)	0 (0%)	1 (1.3%)	0.058 ^†^
**Criminal proceedings**	10 (4.5%)	0 (0%)	0 (0%)	0 (0%)	0 (0%)	0.044
**Concerns about image or manipulation of image**	1 (0.4%)	6 (8.3%)	0 (0%)	0 (0%)	3 (3.8%)	0.002 ^†^
**Concerns about referencing or attributions**	4 (1.8%)	2 (2.8%)	2 (7.7%)	0 (0%)	2 (2.5%)	0.338	
**Error in text**	2 (0.9%)	1 (1.4%)	1 (3.8%)	0 (0%)	2 (2.5%)	0.595
**Copyright claim**	1 (0.4%)	1 (1.4%)	0 (0%)	1 (2.9%)	3 (3.8%)	0.226
**Lack of Institutional Review Boards (IRB) or Institutional Animal Care and Use Committee (IACUC) Approval **	1 (0.4%)	1 (1.4%)	0 (0%)	1 (2.9%)	3 (3.8%)	0.226
**Lack of approval from third party**	1 (0.4%)	1 (1.4%)	0 (0%)	1 (2.9%)	2 (2.5%)	0.479
**Data fabrication or falsification**	1 (0.4%)	1 (1.4%)	0 (0%)	0 (0%)	1 (1.3%)	0.827
**Conflict of interest**	0 (0%)	0 (0%)	1 (3.8%)	1 (2.9%)	1 (1.3%)	0.071
**No informed consent**	0 (0%)	0 (0%)	0 (0%)	0 (0%)	3 (3.8%)	0.500 ^†^
**Non-payment of fees**	2 (0.9%)	0 (0%)	0 (0%)	0 (0%)	0 (0%)	0.751
**Lack of approval from sponsoring company or institution**	1 (0.4%)	0 (0%)	0 (0%)	1 (2.9%)	0 (0%)	0.264

†The results of Fisher’s exact test; other P values are the results of Chi-square test.

The data from 481 retracted articles were reviewed, and 436 biomedical articles met the inclusion criteria. Among these, 223 were from Iran, 35 from Saudi Arabia, 26 from Pakistan, 72 from Egypt, and 80 from Turkey. Supplementary Table-1 presents retraction frequencies from 2010 to 2019.

Across the five countries, the most frequent causes for retraction were text plagiarism (119; 27.3%), article duplication (116; 26.6%), authorship issues (113; 25.9%), and fake peer review (105; 24.1%), detailed in Table-1.

Significant differences were found between Iranian authors and others in retractions because of authorship issues, fake peer review, authors' misconduct, and criminal proceedings (P<0.001, P<0.001, P<0.001, P=0.044, respectively). Iranian and Egyptian authors also differed significantly from others in retractions related to concerns about data, errors in data, or unreliable data (P<0.001), though not significantly from each other (P=0.285). Additionally, Egyptian authors differed significantly from others in concerns about images or manipulation of images (P=0.002).

Of the retracted articles, 354 were indexed in Scopus, 282 in Clarivate Analytics, 254 in MEDLINE, and 149 in PMC. In Iran, Egypt, Saudi Arabia, and Turkey, most were found in Scopus; in Pakistan, Scopus and PMC were equally common (Supplementary Table-2).

Across the five countries, the most retracted articles belonged to biology, biochemistry, medicine-oncology, medicine-cardiology/cardiovascular, medicine-surgery, and medicine-pathology. In Iran, biology (94), biochemistry (34), medicine-pathology (33), oncology (32), zoology (23), and cardiology/cardiovascular (20) had the highest counts. In Egypt, biology (24), pharmacology (14), biochemistry (13), obstetrics & gynecology (11), and surgery (10) led. Pakistan recorded the most in biochemistry and zoology (6 each), followed by biology (4). In Saudi Arabia, biochemistry (8), biology, dentistry, and biostatistics/epidemiology (7 each) ranked highest. In Turkey, biology (28), cardiology/cardiovascular (18), surgery (17), and biochemistry (16) had the most retractions.

## Discussion

The results of our study indicated that text plagiarism, article duplication, authorship issues, and fake peer reviews as the leading causes of retraction across Iran, Saudi Arabia, Pakistan, Egypt, and Turkey.

Among the retracted biomedical articles analyzed, Iran, Egypt, Turkey, Saudi Arabia, and Pakistan showed notable variation in retraction counts. Iran alone accounted for approximately 43% of biomedical publications in the Eastern Mediterranean region between 2004 and 2018, according to WHO and Scopus data [10], which may partially explain its high retraction rate. Although Egypt (14%), Saudi Arabia (11%), and Pakistan (8%) also contribute significantly to the region’s biomedical research output [10], the large gap with Iran may suggest deeper systemic issues, such as academic pressure, weak oversight, or heightened journal scrutiny. These patterns should be interpreted within the broader structural context of the Eastern Mediterranean region, where rapid growth in biomedical publications, limited research oversight, and resource constraints may increase vulnerability to misconduct. Language barriers, particularly the non-native use of English, can affect manuscript preparation and peer review quality. Furthermore, cultural factors, like seniority-based authorship and pressure to publish, may also shape retraction trends.

Similar to our findings, some research has listed detailed reasons for article retraction without using the terms "honest errors" or "scientific misconduct". For example, Dal-Ré used Retraction Watch and found that authorship problems and duplication were the main reasons for retraction [11]. Other studies found honest errors as the primary reason, followed by plagiarism and duplication, though the total number of retractions due to plagiarism and duplication exceeded honest errors [12][13].

Some reported higher retraction rates for honest errors than for misconduct [14]. Since our goal was to report all exact reasons, we did not categorize them into "honest errors" and "scientific misconduct". However, the four common reasons in our study are considered scientific misconduct. This discrepancy may stem from differences in database selection, classification criteria, or time frame.

As shown in our study, the most frequent reasons for article retraction in Iran were fake peer review, authorship issues, plagiarism, article duplication, and author misconduct. In 2016, Springer Nature retracted 58 articles by Iranian authors, citing plagiarism, peer review manipulation, and falsified authorship [15][16].

Talebi described how authors may list a prominent researcher with a fake email in the "Suggested Reviewers" section to manipulate the review process [17]. Ghorbi et al. also identified fake peer review and plagiarism as key issues, suggesting that weak editorial oversight contributes to retractions [18]. Compared to other countries, Iran shows a higher incidence of such misconduct, likely driven by aggressive publication incentives and gaps in journal oversight, which patterns also seen in China, South Korea, and Pakistan [19].

Authorship issues were a frequent cause of retraction in Iranian articles, often rooted in cultural practices like guest authorship, where senior researchers are listed regardless of contribution, a norm some academics view as ethical [20].

Our findings showed that the most retracted articles were indexed in the Scopus, Clarivate Analytics, MEDLINE, and PMC databases, respectively. It can be concluded that most of the articles have been retracted from reputable journals, indicating that misconduct is not limited to low-quality publications. Instead, this reflects increased editorial vigilance and evolving standards of research integrity [2].

A comparison with non-regional countries highlights striking differences in both volume and nature of retractions. China and the United States lead globally in retracted publications, often due to data manipulation, image issues, and fraudulent peer review. While Iran shows a high retraction rate relative to its regional output, countries like India, Japan, and Italy also face notable challenges [21][22]. These findings highlight structural problems in research quality control that differ based on geographical location, economic development, and cultural models, emphasizing the need for improved scientific integrity measures globally.

Retraction rates are notably higher in biomedicine than in other fields [23][24]. Our cross-country analysis found biology and biochemistry most affected, likely due to their interdisciplinary scope and methodological complexity. Pathology and oncology often appeared as secondary fields. These patterns reflect global trends, where cell and cancer biology show high retraction rates driven by ethical problems and data issues [25].

One strength of this study is the simultaneous review of five databases over a nine-year period, along with its multi-country scope, focusing on nations with high publication rates in the region. However, several limitations should be noted. First, we did not calculate the ratio of retracted articles to total publications per country due to challenges in retrieving accurate counts based on first-author affiliation. Database searches by country name include all affiliated authors, while our study focused solely on the first author. Second, using the Retraction Watch database may lead to issues with how retractions are labeled or recorded. Future studies should check data across different sources. Third, the selected time frame may limit generalizability, especially given the surge in retractions during the COVID-19 era. This period was chosen to avoid pandemic-related anomalies, but newer trends warrant investigation. Finally, our first-author focus may exclude retracted articles involving co-authors from the selected countries. We recommend that future studies include all author affiliations to enable a more comprehensive national-level analysis.

## Conclusion

Plagiarism, article duplication, authorship disputes, and fake peer reviews emerged as the primary reasons for retraction in Iran, Egypt, Pakistan, Saudi Arabia, and Turkey, with Iran accounting for the highest number of retracted articles. Notably, many of these retractions occurred in journals indexed by reputable systems, showing that even trusted platforms need careful monitoring. This study makes a distinctive contribution by conducting a multi-country analysis centered on the Eastern Mediterranean region and Turkey, using five major databases over a nine-year period. This comprehensive approach helped us find and classify retracted articles more thoroughly than studies that rely on just one source.

Future research should pursue longitudinal designs to explore evolving retraction patterns, including the time lag between publication and retraction. Calculating retraction-to-publication ratios using full author affiliation data would offer deeper insights into national research vulnerabilities. At the policy level, reforms such as strengthening peer review protocols, enhancing editorial oversight, and implementing ethics training for researchers and editors are key steps to support research integrity in the region.

## Conflict of Interest

There are no conflicts of interest.
